# Effects of Transcranial Direct Current Stimulation on Information Processing Speed, Working Memory, Attention, and Social Cognition in Multiple Sclerosis

**DOI:** 10.3389/fneur.2020.545377

**Published:** 2020-10-15

**Authors:** Christina Grigorescu, Moussa A. Chalah, Jean-Pascal Lefaucheur, Tania Kümpfel, Frank Padberg, Samar S. Ayache, Ulrich Palm

**Affiliations:** ^1^Department of Psychiatry and Psychotherapy, Klinikum der Universität München, Munich, Germany; ^2^EA 4391, Excitabilité nerveuse et thérapeutique, Université Paris-Est-Créteil, Créteil, France; ^3^Service de Physiologie-Explorations Fonctionnelles, Hôpital Henri-Mondor, Assistance Publique-Hôpitaux de Paris, Créteil, France; ^4^Institute for Clinical Neuroimmunology, Klinikum der Universität München, Munich, Germany; ^5^Medical Park Chiemseeblick, Bernau, Germany

**Keywords:** tDCS, social cognition, theory of mind, faux pas test, N-back test, attention, working memory, information processing speed

## Abstract

Multiple Sclerosis (MS) is a chronic inflammatory disease of the central nervous system. Cognitive impairment occurs in 40–65% of patients and could drastically affect their quality of life. Deficits could involve general cognition (e.g., attention and working memory) as well as social cognition. Transcranial direct current stimulation (tDCS), is a novel brain stimulation technique that has been assessed in the context of several neuropsychiatric symptoms, including those described in the context of MS. However, very rare trials have assessed tDCS effects on general cognition in MS, and none has tackled social cognition. The aim of this work was to assess tDCS effects on general and social cognition in MS. Eleven right-handed patients with MS received two blocks (bifrontal tDCS and sham, 2 mA, 20 min, anode/cathode over left/right prefrontal cortex) of 5 daily stimulations separated by a 3-week washout interval. Working memory and attention were, respectively, measured using N-Back Test (0-Back, 1-Back, and 2-Back) and Symbol Digit Modalities Test (SDMT) at the first and fifth day of each block and 1 week later. Social cognition was evaluated using Faux Pas Test and Eyes Test at baseline and 1 week after each block. Interestingly, accuracy of 1-Back test improved following sham but not active bifrontal tDCS. Therefore, active bifrontal tDCS could have impaired working memory via cathodal stimulation of the right prefrontal cortex. No significant tDCS effects were observed on social cognitive measures and SDMT. Admitting the small sample size and the learning (practice) effect that might arise from the repetitive administration of each task, the current results should be considered as preliminary and further investigations in larger patient samples are needed to gain a closer understanding of tDCS effects on cognition in MS.

## Introduction

Multiple sclerosis (MS) is a chronic inflammatory autoimmune disease of the central nervous system, characterized by demyelination, neurodegeneration and synaptopathy (1, 2). It occurs in around 2.3–2.5 million around the globe and is the most common reason of non-traumatic disability in young people (3). Patients with MS (PwMS) could suffer from several symptoms including sensorimotor deficits, cerebellar symptoms, fatigue, as well as emotional, cognitive, and behavioral manifestations (4). Cognitive impairment occurs in 40–65% of patients at one point in their lifetime, could appear in early stages of MS (5, 6) and has a drastic impact on patients' quality of life as well as their daily activities. Cognitive impairment could affect general cognition, such as learning and memory, attention (i.e., information processing speed (IPS), complex, divided and selection attention), language, perceptual-motor and executive functions, as well as social cognition (7–10).

Memory and IPS are among the most deficient cognitive domains in MS (11). Memory impairment occurs in up to 40–65% of patients, with working and long-term memory being importantly affected in the context of MS (12, 13). IPS deficit could be observed in 20–30% of PwMS (9, 13, 14). It is related to decreased neuronal conduction speed secondary to demyelination, and can halt the individual's ability to complete tasks and cope with demanding everyday life requirements (13, 15).

Besides general cognition, there was a recent growing interest to assess the involvement of social cognition in the process of MS [for reviews see (10)]. Social cognition can be seen as mental operations put into action during social interactions, including perception, interpretation, and generating responses to the intentions, dispositions, and behaviors of others (16). Social cognition entails the individual's ability to (a) recognize emotions from social stimuli cues, (b) infer others' mental state based on their intentions, thoughts and beliefs [i.e., cognitive theory of mind (ToM)], and their emotions and desires (i.e., affective ToM), and (c) empathize with others (10, 17, 18). Social cognition influences the relationship with friends, family, colleagues, and strangers. Thus, it has a high impact on peer support which is a relevant factor for good quality of life and coping with everyday life difficulties, a coping that is particularly important in patients suffering from a chronic and debilitating disease such as MS (19). There is evidence that PwMS show considerable social cognitive deficits that are at the origin of additional burden in this population (10, 20, 21).

From a neurobiological perspective, neuroimaging studies have explored the structural and functional correlates of cognitive impairment in MS. Some studies linked cognitive impairment to pathologies involving the frontal, parietal, temporal and thalamic regions [For reviews see (22)]. It is noteworthy that the frontal lobe constitutes a carrefour for several cognitive tracts, and many studies have linked cognitive impairment in MS with abnormalities involving the (pre)-frontal cortex and/or its connectivity [For reviews see (23)].

From a therapeutic perspective, despite the serious impact of cognitive deficits on this population, efficacy of pharmacological and cognitive interventions has not been supported by enough evidence [For reviews see (11)]. Therefore, alternative interventions might have their place in this context, and their effects merit to be explored. Recently, non-invasive brain stimulation (NIBS), notably transcranial direct current stimulation (tDCS), has shown promising results in the treatment of MS-related symptoms, with most of studies focusing on MS fatigue (4, 24). However, tDCS effects on MS-related cognitive deficits have been rarely addressed. Positive effects have been suggested by few trials that have combined this technique with cognitive training (25, 26), or by some case reports [(10, 27–29), for a review see: 4].

The present report is part of a randomized double-blind sham-controlled cross-over study designed to assess the effects of anodal bifrontal tDCS on MS fatigue as well as other components of the symptoms cluster (i.e., anxiety and depression) (30). Five consecutive daily tDCS sessions led to acute antifatigue effects and delayed anxiolytic effects that emerged 1 week later (30).

Here, we aimed to study the effect of anodal bifrontal tDCS on general cognition (i.e., attention, working memory and IPS) and social cognition in PwMS. Neuroimaging studies (functional MRI and [^11^C]-raclopride Positron emission tomography performed in healthy individuals) and computational model analysis suggest that bifrontal tDCS could modulate the function of cortico-subcortical circuits [i.e., (31–33)]. In MS, despite the lack of studies assessing tDCS mechanisms of action on cognitive functions, the application of high frequency repetitive transcranial magnetic stimulation (another NIBS technique which is supposed to activate the cortical area in question) over the left prefrontal cortex resulted in a cognitive improvement that was paralleled by an increase in prefrontal functional connectivity (34). Therefore, following the same logic, we hypothesized that enhancing the activity of frontal regions and their connectivity, by applying tDCS, could improve general and social cognitive performance in this clinical population.

## Materials and Methods

### Participants

The study took place at the Department of Psychiatry and Psychotherapy of University of Munich Hospital. Recruitment occurred at the Institute of Clinical Neuroimmunology and Cooperating Neurological Practices. Right-handed patients (age: 18–75 years), with a definite MS diagnosis [according to 2017 revised McDonald criteria; (35)] and low disability [Expanded Disability Status Scale score (EDSS) <6.5; (36)] took part of the study. They had stable treatments (≥ 1 month) and did not suffer from relapses (During the last 2 months), or other relevant neuropsychiatric diseases [inclusion/exclusion criteria details are reported in Chalah et al. (30)]. The local ethics committee approved the study which was conducted in conformity with the declaration of Helsinki. All patients gave written informed consent prior to inclusion. Eleven patients (8 females) participated in the study protocol.

### Evaluation

#### Attention, Working Memory, and Information Processing Speed

The N-Back task is commonly used to assess working memory in MS studies (37, 38). In addition, this task has been widely adopted in tDCS studies that documented working memory improvement in healthy and some neuropsychiatric populations [for review and meta-analysis, please refer to Brunoni and Vanderhasselt (39)]. Among these studies, some have documented improvement in N-Back outcomes following the application of a single session of bifrontal tDCS (anode/cathode over F3/F4) in healthy individuals [*n* = 10, (40)] or depressed patients [*n* = 28, (41)].

N-Back v5 was used in this study. Presentation of visual stimuli and recording of responses were controlled using Presentation Software (Neurobehavioral Systems, Inc., Albany, CA, USA). In this experiment, working memory was evaluated using three difficulty levels, the latter differ in the number of items to memorize (i.e., 0, 1, or 2 items) and refer to as 0-Back, 1-Back, and 2-Back. The 0-Back condition is the easiest one in which the target consisted of any item that matches a pre-specified item, and hence this condition requires sustained attention but no working memory demand (42). The two other conditions are of increasing difficulties and evaluate working memory. The targets of the 1-Back and 2-Back conditions correspond to any item identical to the item presented one trial and two trials back, respectively. For each condition, results are displayed as accuracy and reaction time.

Symbol Digit Modalities Test (SDMT) was used to assess IPS and visuospatial attention (43). This task was adopted because it is easy to use, fast to administer, does not cause any significant amount of stress for patients, and is considered a sentinel test to assess cognitive status in PwMS (44). In addition, this test was previously employed in the only two available studies assessing tDCS effects on cognition in PwMS (25, 26). A key that pairs single digits with nine symbols is presented, and the individual is asked to fill rows containing only symbols by matching them with the correct numbers according to the key. The score corresponds to the total number of correct answers that the individual obtains in 90 s. The same versions of SDMT and N-Back were used during all the evaluations.

#### Social Cognition

Social cognition was assessed by means of Reading the Mind in the Eyes Test (Eyes Test) and Faux Pas Test, which, respectively, assess the affective and cognitive components of ToM (45–47). Eyes Test is a non-verbal test that assesses the affective component of ToM and consists of 36 eye pictures of actors and actresses expressing several emotional states and the patient is asked to interpret the social sign hidden in the pair of eyes. Initially developed for autism disorders, other psychiatric and neurological patients were found to poorly perform on this test (10, 45, 47, 48). Eyes Test score is calculated by summing up all individual items, with higher scores indicating better skills.

Faux Pas Test is a verbal test that measures cognitive ToM (46). The test assesses the ability of an individual to detect a “faux pas” which could occur “when a speaker says something without considering if it is something that the listener might not want to hear or know, and which typically has negative consequences that the speaker never intended” (46). The test consists of reading faux pas stories and control stories for individuals. Afterwards, the individual is assessed for their capacity to understand inappropriateness, intentions, and false beliefs. For the Faux Pas Test, individual scores given for short stories are summed up. The higher the score the better the performance is. The same versions of Eyes Test and Faux Pas Test were used during all the evaluations.

### Transcranial Direct Current Stimulation

**Figure 1:**
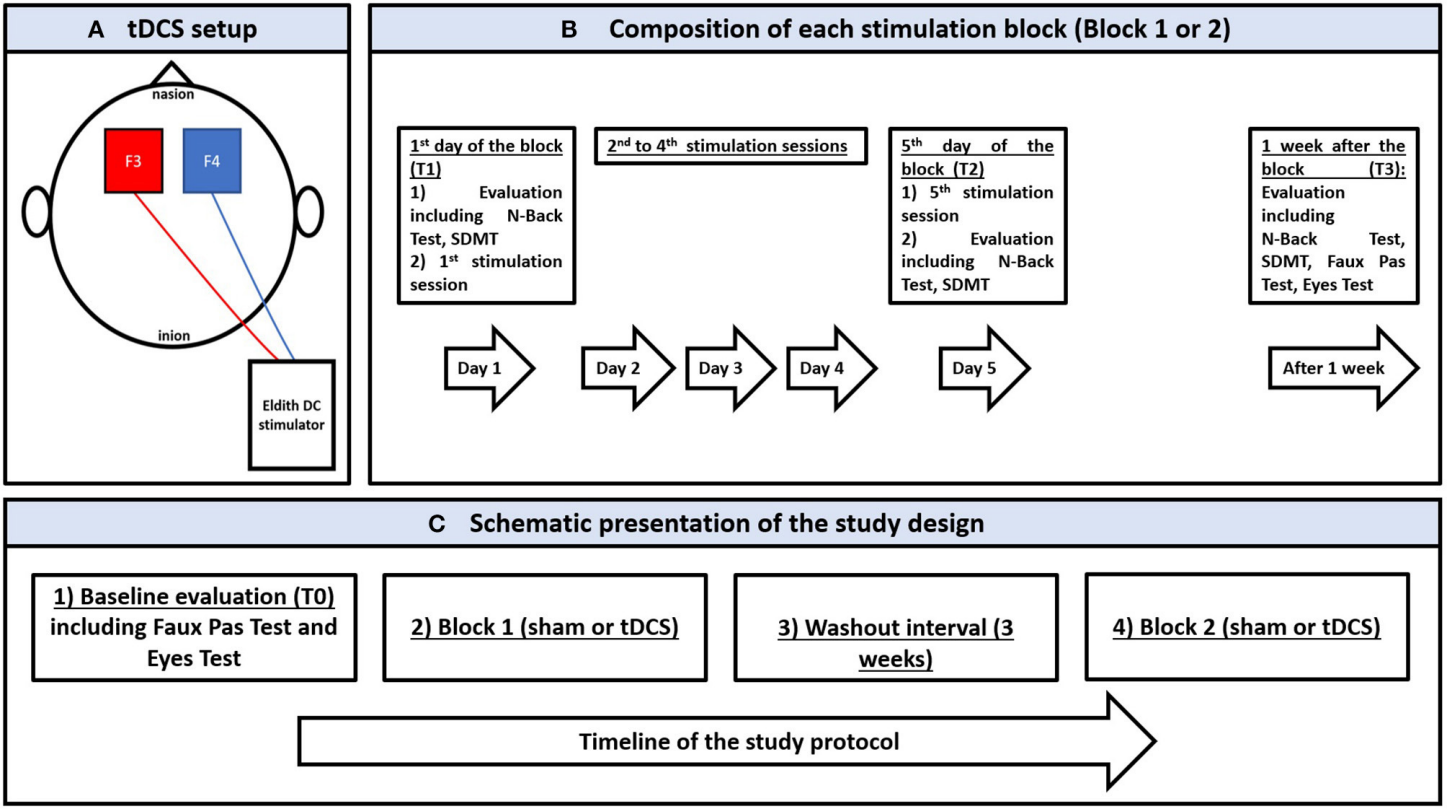
**(A)** schematic presentation of the transcranial direct stimulation setup showing the anode (in red) and the cathode (in blue) over F3 and F4, respectively, according to the 10–20 international electroencephalographic system for electrode positioning; the electrodes are connected to a battery-driver direct current stimulator. **(B)** schematic presentation of a stimulation block (sham or active) showing the stimulation and evaluation sessions between the 1st day of stimulation and 1 week after the last stimulation. **(C)** schematic presentation of the study protocol showing the chronological order of experimentation. SDMT: Symbol Digit Modalities Test.

A weak electric current (i.e., 2 mA) is applied via a CE-certified battery driven stimulator (Eldith DC stimulator, NeuroConn, Ilmenau, Germany) and two saline-soaked sponge electrodes (5 cm x 7 cm) fixed by rubber bands with the anode and cathode over the left and right dorsolateral prefrontal cortices (DLPFC) (F3 and F4 according to the EEG 10–20 system) (49). tDCS setup is presented in Figure 1A. Patients were randomly assigned to receive active and sham tDCS blocks in a cross-over design. Each block consisted of five consecutive daily sessions, with 20 min per session (Figure 1B). Blocks were separated by a 3-week washout interval. For the active condition, current ramping up and down was done over 15 s at the beginning and end of each session, respectively, separated by 20 min stimulation. For sham, the same pattern of ramping was performed with only 30 s of stimulation aiming to simulate the cutaneous sensations obtained with active tDCS (50). tDCS parameters (i.e., current intensity, polarity, sessions number and duration) were chosen according to previous works performed in MS and other clinical populations [for reviews see (4, 24)].

### Study Protocol

Patients were evaluated for eligibility. In case of eligibility and agreement to participate in the study, patients gave their informed written consent, underwent a baseline evaluation (T0), and were randomized to receive tDCS blocks. Allocation to start with an active or sham treatment was performed by a computerized random generator.

In each block, tDCS was applied from the first day (T1) to the 5^th^ day (T2) while patients were at rest, sitting in a comfortable chair in a quiet room.

N-Back Test and SDMT were performed at T1 and T2, and 1 week after each block (T3). Given the potential susceptibility of social cognitive measures to practice effects (51), as well as the absence of learning effect and the acceptable test-retest reliability that are reported when repeating these measures few weeks after a first evaluation (i.e., (52, 53)), Eyes Test and Faux Pas Test were assessed at T0 and T3 of each block. Figure 1C provides a schematic presentation of the study design.

### Statistical Analysis

Statistical analyses were performed using SPSS software (Version 24.0, Armonk, NY: IBM Corp.) and all measures were compared between active and sham conditions. Since not all data followed normal distribution according to Kolmogorov-Smirnov test, a non-parametric analysis of variance was run for group comparison using Friedman's test and *post-hoc* Dunn's test, and took into consideration the groups (active vs. sham) and the time points (T1, T2, and T3 for N-back Test and SDMT; T0 and T3 in the case of Eyes Test and Faux Pas Test). For Friedman's test, estimation of effect size was based on Kendall's *W* coefficient of concordance (54), with effect size being considered small (< 0.3), moderate (0.3–0.5) and large (≥ 0.5). To test for a possible carry-over effect (i.e., effects from the first block that could persist in the second block), Wilcoxon's test was run on data obtained prior to each stimulation block (pre-active vs. pre-sham). In addition, to test for possible learning that could result from repeated exposure to the same tests (i.e., practice effect), the patients' scores on each test were grouped according to the chronological order of evaluations (regardless which stimulation condition was administered first), and were compared using Friedman's test and *post-hoc* Dunn's test. For all tests, significance was set at 0.05. Data are presented as mean ± SD.

## Results

### Sociodemographic and Clinical Data

The mean patients' age was 43.91 ± 9.69 years (age range 26–57 years). Mean disease duration was 75.64 ± 45.97 months. Mean EDSS was 3.14 ± 1.31. Ten patients had a relapsing-remitting MS and 1 patient had a secondary-progressive MS. Nine patients were receiving immunomodulatory treatments. tDCS was well-tolerated and there were no serious adverse effects at any time. tDCS safety and patients' clinical discomfort did not significantly differ according to the stimulation condition (30).

### Cognitive Data

No significant differences were observed in cognitive scores obtained prior to the active and sham interventions (Wilcoxon's test).

#### Attention, Working Memory and Information Processing Speed

**Figure 2:**
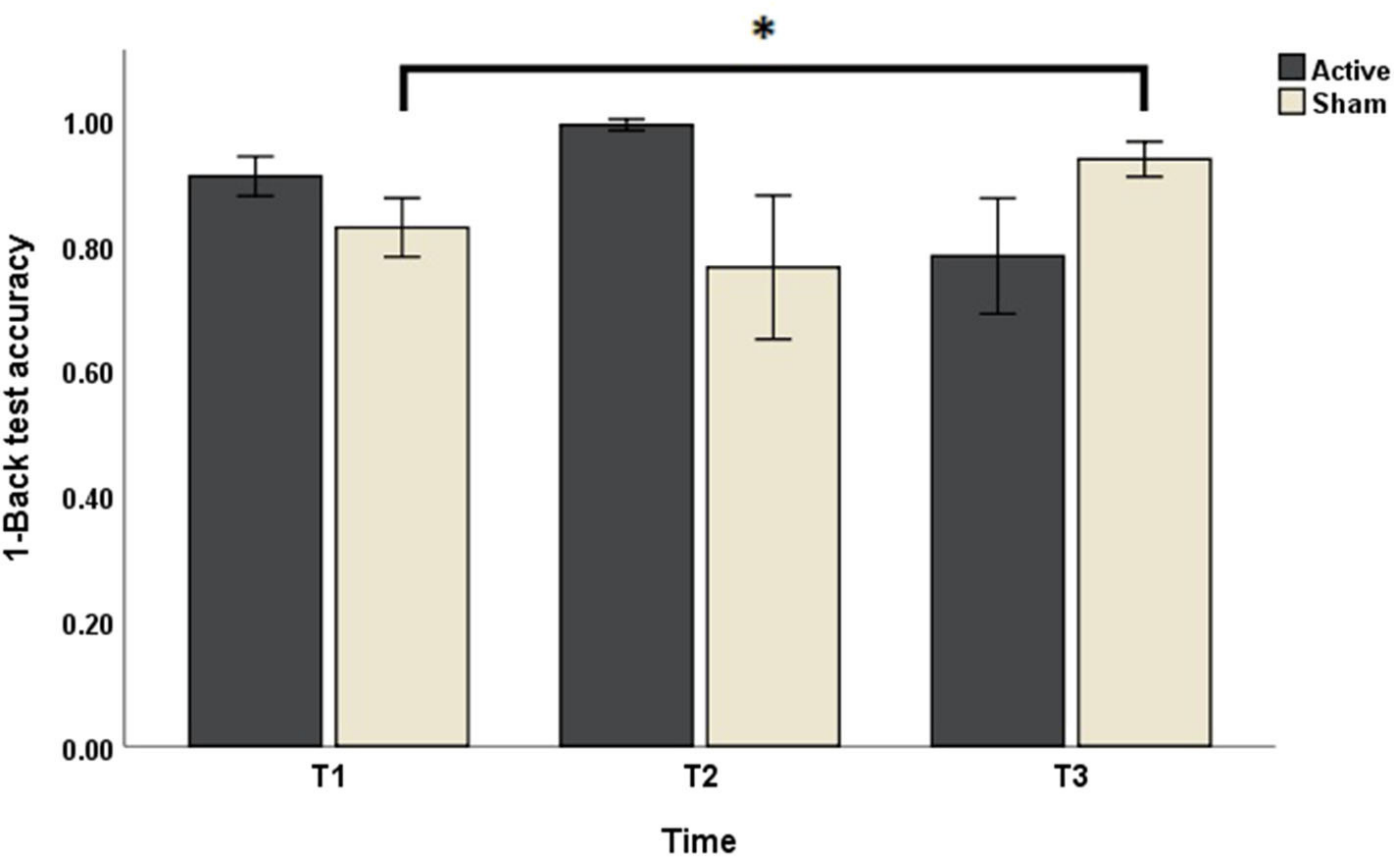
The effects of sham and active stimulation on 1-Back test accuracy. T1, T2, and T3: day 1, day 5, and 1 week after each stimulation block, respectively. *p < 0.05.

Concerning the outcomes obtained with N-Back test, Friedman's test of differences among repeated measures rendered a *X*^2^ value of 13.14 which was only significant in the case of 1-Back accuracy (*df* = 5; *p* = 0.022). *Post-hoc* analysis revealed significant effects obtained, not right after sham intervention (T1: 0.83 ± 0.16 vs. T2: 0.76 ± 0.38; *p* > 0.05), but rather 1 week later (T3: 0.94 ± 0.09, *p* < 0.05) (Figure 2). Effects of active intervention did not reach statistical significance right after the intervention (T1: 0.91 ± 0.10 vs. T2: 0.99 ± 0.03; *p* > 0.05) or 1 week later (T3: 0.78 ± 0.31, *p* > 0.05) (Table 1). 1-Back accuracy at T1 did not differ between active and sham conditions (*p* > 0.05). Results of N-Back outcomes are summarized in Table 1.

**Table 1:** The effects of sham and active stimulation on N-Back Test (0-Back, 1-Back and 2-Back) and Symbol Digit Modalities Test (SDMT).

**Studied outcomes**	**Friedman's test *p*-value**	**Kendall's *W***	**T1**	**T2**	**T3**	**Dunn's test *p*-value**	**T1**	**T2**	**T3**	**Dunn's test *p*-value**
			**Active stimulation**				**Sham stimulation**			
0-Back accuracy	0.157	0.145	0.84 ± 0.35	0.90 ± 0.30	–	>0.05	0.77 ± 0.39	0.99 ± 0.03	–	>0.05
				–	0.99 ± 0.03	>0.05		–	0.98 ± 0.06	>0.05
0-Back RT (ms)	**0.044**	0.207	541.47 ± 200.68	498.04 ± 186.69	–	>0.05	445.64 ± 244.80	514.76 ± 81.84	–	>0.05
			541.47 ± 200.68	–	558.30 ± 85.34	>0.05	445.64 ± 244.80	–	514.15 ± 76.62	>0.05
1-Back accuracy	**0.022**	0.239	0.91 ± 0.10	0.99 ± 0.03	–	>0.05	0.83 ± 0.16	0.76 ± 0.38	–	>0.05
			0.91 ± 0.10	–	0.78 ± 0.31	>0.05	0.83 ± 0.16	–	0.94 ± 0.09	**<0.05**
1-Back RT (ms)	0.628	0.063	625.84 ± 106.43	600.86 ± 98.38	–	>0.05	633.72 ± 114.93	599.26 ± 222.83	—	>0.05
			625.84 ± 106.43	–	570.47 ± 217.95	>0.05	633.72 ± 114.93	–	567.58 ± 62.49	>0.05
2-Back accuracy	0.235	0.124	0.68 ± 0.15	0.74 ± 0.24	–	>0.05	0.68 ± 0.17	0.67 ± 0.28	–	>0.05
			0.68 ± 0.15	–	0.78 ± 0.16	0.05	0.68 ± 0.17	–	0.77 ±0.12	>0.05
2-Back RT (ms)	0.732	0.051	717.59 ± 93.62	741.46 ± 154.31	–	>0.05	754.33 ± 174.91	808.40 ± 193.70	–	>0.05
			717.59 ± 93.62	–	708.07 ± 144.24	>0.05	754.33 ± 174.91	–	696.64 ± 110.46	>0.05
SDMT	0.063	0.191	49.73 ± 12.28	54.00 ± 14.07	–	>0.05	48.55 ± 10.89	50.45 ± 10.98	–	>0.05
			49.73 ± 12.28	–	55.64 ± 11.63	>0.05	48.55 ± 10.89	–	52.73 ± 9.47	>0.05

*T1, T2, and T3: day 1, day 5, and 1 week after each stimulation block, respectively. RT, reaction time. Significant p-values are bolded*.

Concerning SDMT scores, Friedman's test rendered a *X*^2^ value of 10.48 which was non-significant (*df* = 5; *p* = 0.063) (Table 1). Kendall's *W* coefficient of concordance was <0.3 (small effect size) for all outcomes (details are mentioned in Table 1).

When studying the learning effect, a significant learning effect was observed regarding SDMT scores (Friedman's test *p* < 0.001). *Post-hoc* analysis revealed significant increases in SDMT scores starting the 4^th^ evaluation (*p* < 0.05).

No significant learning effect was observed regarding 0-Back accuracy (Friedman's Test *p* = 0.069), 0-Back reaction time (Friedman's Test *p* = 0.391), 1-Back accuracy (Friedman's Test *p*=0.191), 1-Back reaction time (Friedman's Test *p*=0.582), 2-Back accuracy (Friedman's Test *p* = 0.169) or 2-Back reaction time (Friedman's Test *p* = 0.652).

#### Social Cognition

Friedman's test showed a *X*^2^ value of 1.81 for the Eyes Test which was non-significant (*df* = 2, *p* = 0.406). The same applies to the Faux Pas Test where no significant difference was observed following sham and active conditions (Friedman's Test *X*^2^= 2.61; *df* = 2; *p* = 0.272). Table 2 presents the different scores obtained at each time point. Kendall's *W* coefficient of concordance was <0.3 (small effect size) for both tests (details are mentioned in Table 1). No significant learning effect was observed for the Eyes Test (Friedman's Test *p* = 0.803) or Faux Pas Test (Friedman's Test *p* = 0.307).

**Table 2:** The effects of sham and active stimulation on Eyes Test and Faux Pas Test scores.

			**Time points**			
**Studied outcomes**	**Friedman's test *p*-value**	**Kendall's *W***	**Baseline**	**Post-active stimulation**	**Post-sham stimulation**	**Dunn's test *p*-value**
Eyes test	0.406	0.082	24.09 ± 4.97	23.27 ± 3.64	–	>0.05
			24.09 ± 4.97	–	24.73 ± 4.52	>0.05
Faux pas test	0.272	0.118	22.18 ± 3.71	20.55 ± 3.91	–	>0.05
			22.18 ± 3.71	–	19.45 ± 4.01	>0.05

*All differences were not significant at 0.05*.

## Discussion

This study evaluated tDCS effects on general cognition (particularly attention, working memory, and IPS), as assessed by N-Back Test and SDMT, and social cognition, according to Faux Pas and Eyes Tests, in patients with MS. The main finding of this work consisted of a significant delayed improvement in 1-Back accuracy obtained 1 week after sham intervention. This outcome was not found with active stimulation. Neither intervention had significant effects on the remaining outcomes.

### tDCS and Attention, Working Memory, and Information Processing Speed

Cognitive performance (accuracy in 1-Back Test) interestingly improved after sham, but not after tDCS condition. A systematic review reports mixed effects of anodal tDCS on working memory performance (55). Our results are in line with previous findings on this matter (56–58). For instance, in studies involving healthy participants, anodal bifrontal tDCS hampered the accuracy (58). In addition, in a study involving patients with major depressive disorder, the accuracy on procedural or implicit learning task improved following sham but not active stimulation as seen in our present work (57). The authors concluded that bifrontal tDCS prevented implicit learning in their cohort.

Here, it is worth noting that the negative findings of the current study could be partly attributed to the low statistical power of our sample, although in some studies using similar sample size (*n* = 10), a single 10 min session of bifrontal tDCS was able to improve N-Back outcomes in healthy volunteers (40). In this context, it is important to mention that tDCS response might differ between the healthy and diseased brain, as well as across clinical populations. In fact, Hill and colleagues have reported a significant improvement in offline working memory tasks in healthy but not in neuropsychiatric cohorts (55). Relative to healthy individuals, MS patients might suffer from baseline cortico-subcortical abnormalities and changes in regional connectivity, which might have compromised the emergence of robust changes.

Another possible explanation of our negative findings could be the placement of the reference electrodes. In fact, in bifrontal montage, the reference electrode is over the right DLPFC. It seems that this have resulted in cathodal stimulation of this area, and hence an inhibition of cognitive processes to which it contributes. Here, it is worth noting that the right DLPFC is an important carrefour that gets activated during visual working memory tasks (59–62), and a damage of this area could impair working memory as demonstrated in lesion studies (61, 63, 64). Therefore, in our work, the relative improvement of working memory obtained following sham intervention would indirectly hint toward an impairment of this cognitive ability following active condition. An impairment that is probably due to the inhibition of right DLPFC by cathodal stimulation.

Several works have highlighted the role of the right DLPFC in working memory (65–67), among which some consisted of tDCS works that documented an improvement of working memory when placing the anode over this area in healthy populations [cathode: over left cheek in Wu et al. (68); over the contralateral supraorbital area in Giglia et al. (69); over Cz in Bogdanov and Schwabe (70); and over F3 in Nissim et al. (33)]. Therefore, it would be interesting in future works to set the anode over this area (F4) and determine the optimal return electrode to ameliorate working memory.

Besides working memory, attention and IPS do not seem to be affected by tDCS in this study, although the observed learning effect might have prohibited observing such changes. The current findings are consistent with previous works that reported lack of tDCS effects on attention or IPS when applied over 3–5 consecutive days in MS (10, 71, 72). Conversely, few trials suggested the add-on benefits of 10 sessions of anodal prefontal tDCS stimulation when combined with cognitive training (25, 26). Moreover, positive effects were reported in few case reports that applied 14–40 anodal tDCS sessions over the left prefrontal cortex (10, 27, 28). Therefore, longer stimulation duration and combination with cognitive training might improve cognition.

### tDCS and Social Cognition

Social cognition did not significantly improve following tDCS. To the best of our knowledge, this is the first study to address the effects of tDCS on social cognition in MS. The idea of modulating social cognition by tDCS targeting the prefrontal cortex stems from studies showing the involvement of this region in social cognition (73), and its relationship with social cognitive deficits in MS (10). Unlike our study, 12 sessions of bifrontal tDCS improved social cognition in depressed patients (74). Moreover, a single session of anodal tDCS ameliorated social cognitive measures in healthy individuals [left prefrontal anodal stimulation, right frontopolar cathode; (75)], as in patients with neurodegenerative diseases [medial prefrontal anodal stimulation; in frontotemporal dementia (76) and in Parkinson's disease (77)]. It is noteworthy that, with regards to social cognition, a hemispheric asymmetry seems to exist for some processes, and the right cerebral hemisphere appears to be important for social cognitive processes (78, 79). Therefore, as suggested for working memory, it would be of interest when targeting social cognition to test the application of anodal tDCS over the right DLPFC. However, an attention should be paid when selecting the other electrode (the reference electrode) since an anodal F4/cathodal F3 setup was found in few works to negatively affect some social cognitive aspects such as adopting others' perspective (80) or empathy for pain (81).

Future studies would also benefit from increasing the number of stimulation sessions and investigating the utility of targeting other cortical areas, such as the right temporoparietal junction and the ventromedial prefrontal cortex (82–85).

### Limitations and Perspectives

This study has several limitations. First, admitting the small sample size and the small effect size estimates (all below <0.3), this work should be considered a pilot study with non-definite preliminary results. Larger studies are needed to further explore these findings. Second, the cross-over design could have provoked overlapping effects. The wash-out interval of 3 weeks may be too short to prevent the effects from the first block to interfere with the second stimulation block.

Third, a limitation might arise from the employed tools to evaluate cognition. Although this study included cognitive measures that are widely used in MS research, the use of the same tests several times across the study is a key point to consider since it may imply a potential practice effect (86, 87), as was observed with SDMT scores in this work. Future studies would benefit from employing alternate forms of the cognitive tasks at each evaluation. In that context, alternate forms of SDMT have been proposed in MS studies; they are reliable and equivalent in difficulty which could help overcoming the practice effect when considering cognitive outcomes in future tDCS trials (88, 89). Similarly, alternate forms of memory tests (including N-Back test) using the same set of stimuli with different order or composition have also been suggested (90, 91). Moreover, retesting in social cognition may be problematic. Although the tests employed in this work stand among the most adopted in the literature (i.e., Eyes Test and Faux Pas Test), no alternate forms seem to exist for these tests. Thus, employing social cognitive tasks that are available in multiple forms (e.g., The Assessment of Social Inference Test, the Hinting Task) could help avoiding the practice effect [for reviews see (92)]. However, when choosing to employ the same vs. alternate forms of a task, it is also important to consider the possibility of statistically accounting for the practice effect related to the repeated administration of the same task as well as the challenges related to the use of alternate forms, namely the number of required forms that increases with the number of testing points and the differences in task difficulty across the different forms (87).

Fourth, although the evaluation of IPS and sustained attention included tasks that are considered simple, insight from neuroimaging studies suggest that some of these tasks are complex and recruit cortico-subcortical networks [(93–95). Therefore, including a simpler task might have been more sensitive to detect subtle tDCS effects; this could have been done using a simple reaction time task which for example requires the individual to press a button as soon as a single stimulus appears in the center of a computer screen (96). Besides the tasks' complexity, another drawback is related to the choice of social cognition tools. Social cognition is a complex construct of multiple components that was assessed by static tasks. Dynamic tasks (i.e., videotapes featuring social scenes) might have better ecological validity (10), and merits to be adopted in tDCS studies on MS. Future studies could also benefit from assessing tDCS effects on other general (i.e., perceptual-motor and executive functions, language) or social cognitive domains (i.e., emotion recognition from facial, vocal or bodily cues, empathic ability).

Finally, as stated above, it would be interesting to test different tDCS variables (i.e., polarity, electrode locations and montage, sessions number and duration, current intensity) in order to determine the optimal parameters to improve cognitive functions in MS. For instance, applying greater tDCS doses (i.e., intensity and duration) and/or combining them with other interventions might lead to synergistic effects. However, repeating the sessions and including patients in a protocol lasting several weeks might be difficult; a home-based and remotely supervised treatment could fill this gap (97).

## Conclusions

This study assessed the effects of five consecutive daily 20 min sessions of bifrontal tDCS on cognition in MS. 1-Back accuracy improved after sham but not after active tDCS. Bifrontal tDCS seems to impair working memory in PwMS. No other significant effects were observed on attention, IPS, or social cognition. A larger patient sample and potentially a longer stimulation interval and follow up could help confirming the current results.

## Data Availability Statement

The raw data supporting the conclusions of this article will be made available to any qualified researcher on request to the corresponding author.

## Ethics Statement

The studies involving human participants were reviewed and approved by Ludwig Maximilian University Munich. The patients provided their written informed consent to participate in this study.

## Author Contributions

MAC, SSA, J-PL, FP, and UP designed the study. Participants were recruited by TK and screened by CG. CG performed data collection. MAC and SSA performed the analysis. The manuscript was drafted by CG, MAC, SSA, and UP. All authors participated to data interpretation, critically revised the manuscript, and approved the final version.

## Conflict of Interest

FP is a member of the European Scientific Advisory Board of Brainsway Inc., Jerusalem, Israel, and has received speaker's honoraria from Mag & More GmbH and the neuroCare Group. His lab has received support with equipment from neuroConn GmbH, Ilmenau, Germany, and Mag & More GmbH and Brainsway Inc., Jerusalem, Israel. SSA declares having received travel grants or compensation from Genzyme, Biogen, Novartis and Roche. UP has a private practice with NeuroCare Group, Munich, Germany. MAC declares having received compensation from Janssen Global Services LLC. TK has received travel expenses and speaker honoraria from Bayer Vital, Teva Pharma, Merck, Novartis Pharma, Sanofi-Aventis/Genzyme, CLS Behring, Roche Pharma and Biogen as well as grant support/donation from Bayer-Schering AG, Novartis and Chugai Pharma. The remaining authors declare that the research was conducted in the absence of any commercial or financial relationships that could be construed as a potential conflict of interest.
